# Effect of β-Caryophyllene on oxidative stress, glucose metabolism in the skeletal muscle of high fat diet and fructose-induced type-2 diabetic adult male rats

**DOI:** 10.6026/97320630019417

**Published:** 2023-04-30

**Authors:** Syamala Devi Bandaru, Manigandan Balraj, Ramya Badrachalam, Vadivel Mani

**Affiliations:** 1 Department of Physiology, Konaseema Institute of Medical sciences and research foundation, Amalapuram, East Godavari Dt-533201, Andhra Pradesh, India; 2 Department of Biochemistry, Sri Manakula Vinayagar Medical College and Hospital, Puducherry - 605107, Tamil Nadu, India; 3 Department of Biochemistry, Konaseema Institute of Medical sciences and research foundation, Amalapuram, East Godavari Dt-533201, Andhra Pradesh, India

**Keywords:** Type-2 diabetes, β-Caryophyllene, High fat diet, skeletal muscle, glucose uptake, glycogen content

## Abstract

Skeletal muscle is where the majority of insulin-stimulated whole-body glucose elimination takes place under normal circumstances. A significant risk factor for metabolic diseases is high dietary fat consumption, which also increases stored fat mass. Natural solutions with anti-diabetic effectiveness and fewer side effects are becoming more popular as a result of the conventional pharmacological treatments' numerous negative side effects and high rates of secondary failure. Cannabis and a variety of culinary herbs and spices may include the naturally occurring sequiterpene β-caryophyllene. Among other things, it has antioxidant, anti-inflammatory, and anti-lipidemic properties. However, it is not yet known how β-caryophyllene affects the uptake and oxidation of glucose. Determining if β -caryophyllene has anti-diabetic properties in type-2 diabetes brought on by a high-fat diet was the objective of the current investigation. A sufficient dose of β-caryophyllene (200 mg/kg b.w.t., orally for 30 days) was given to type-2 diabetic rats fed a high-fat diet and given fructose as an inducer of diabetes to assess its anti-diabetic activity. The treatment of diabetes-induced rats with β -Caryophyllene restored the altered levels of blood glucose, serum insulin as well as the lipid parameters, oxidative stress markers, antioxidant enzymes. Our findings show that β-caryophyllene improves glycemia control by enhancing glucose absorption and oxidation in the skeletal muscle of type-2 diabetic rats. From the present findings, it is evident that β -caryophyllene can be used as an anti-diabetic drug.

## Background:

Diabetes mellitus is a long-term metabolic condition characterized by insufficient insulin production, insulin action resistance, or both. It is associated with disturbances in carbohydrate, lipid and protein metabolism, which leads to hyperglycemia, hyperlipidemia, hyperinsulinemia and hypertension [1]. Diabetes is a leading contributor to conditions including blindness, limb amputation, renal failure, and cardiovascular disorders like heart attack and stroke [2]. Diabetes currently affects about 463 million people globally, and by 2045, that figure will rise to 700 million. Type-2 diabetes comprises 90% around the world and more prevalent when compared with type-1 diabetes [3]. Diet, exercise, and chemotherapy are typically used to treat diabetes mellitus. Due to the widespread adverse side effects and high rates of eventual failure associated with conventional pharmaceutical therapies, natural remedies with anti-diabetic effectiveness and fewer side effects are growing in popularity [4]. Therefore, it's critical to look for more economical, few to no side effects, and more effective anti-diabetic drugs. Preferably, they should come from dietary sources. Many experimental studies have elucidated that flavonoids, terpenoids and other secondary metabolites of plant possess hypoglycemic effects in different experimental models [5] Due to their effectiveness and abundance in plants, trends on assessing the glycemic control effect of herbal and dynamic compound of plants have attracted a lot of attention. β- Caryophyllene is a naturally occurring sequiterpene that can be found in cannabis as well as a variety of cooking herbs and flavors of food. This terpene is abundant in essential oils from hemp, hops, rosemary, black pepper, and cinnamon extracts, which both contain 30% and 17.4% of it, respectively. It has a wide range of biological actions, including antioxidant, anti-inflammatory, and anti-lipidemic properties [6]. It could aid in treating seizures, decreasing cholesterol, and easing anxiety and pain. Being an anti-inflammatory agent, β -caryophyllene protects against oxidative stress and might be a beneficial preventative medication for a variety of medical problems such as liver diseases, renal diseases, liver and gastrointestinal illnesses, and immunological and neurological diseases [6, 7, 8, 9]. In streptozotocin (STZ)-induced diabetic mice, chronic oral treatment with β-caryophyllene decreases glycemia, depressive-like behavior, and neuropathic pain [10]. Furthermore, it has recently been discovered that β-caryophyllene efficiently protects β-cells in Langerhans islets by relieving hyperglycemia by boosting insulin release. Additionally, it protects beta-cells from inflammation feedback from oxidative stress in diabetic rats [11]. It is currently unknown how β-caryophyllene affects the absorption and oxidation of glucose. Therefore, the goal of the current study was to investigate how high-fat diet-induced type-2 diabetes was affected by β- caryophyllene anti-diabetic effects.

## Materials and Methods 

## Animals:

In this study, we used 150-180 day old Wistar strain healthy adult male albino rats. The Institutional Animal Ethics committee (Reg No: 765/03/ca/CPCSEA and approval certificate No IAEC No: 007/2019, dated 04/11/2019) at Meenakshi Medical College and Research Institute, MAHER, Enathur, Kanchipuram, Tamil Nadu-631552, India, approved their treatment in line with national rules and protocols. Animals were housed at a constant temperature (21± 2°C) and humidity (65 ± 5%) with a 12 hour light and 12 hour dark cycle, and fed a normal pelleted food (Lipton India, Mumbai, India) with clean drinking water supplied ad libitum.

## Chemicals:

All chemicals, reagents and metformin used in the present study were of molecular and analytical grade and they were purchased from Sigma-Aldrich Chemical Company, St. Louis, MO, USA and Sisco Research Laboratories, Chennai, India. β- Caryophyllene was purchased from Tokyo Chemicals Industry Co., LTD, Tokyo, Japan. On-Call Plus Blood glucose test strips were purchased from ACON Laboratories, Inc. San Diego, USA.

## Induction of Type-2 Diabetes:

To induce type-2 diabetes in rats for 60 days, a high-fat diet containing 2% cholesterol, 1% cholic acid, 30% coconut oil, 67 percent ordinary rat feed, and 25% fructose through drinking water was given to the rats [12]. Animals were recruited for the experiment if their fasting blood glucose levels were greater than 120 mg/dl after 60 days of measurement. The high-fat diet and sugar feeding were maintained until the study conclusion. Normal pelleted rat feed was provided to control rats and water was freely available.

## Experimental design

The rats were given therapy for a month according to the following experimental design, which was set up. The following groups, each with six rats, were formed from healthy and type-2 diabetic adult male Wistar rats.

Group I: Control (Normal rats).

Group II: Rats were made diabetic (type-2) after feeding high fat diet and fructose through drinking water (30%) for 60 days.

Group III: Type-2 diabetic rats treated orally with β-caryophyllene (200 mg/kg b.wt/day) once in a day, orally for 30 days.

Group IV: Type-2 diabetic rats treated orally with metformin (37) (50 mg/kg, b.wt/day) [13] once in a day, orally for 30 days.

Group V: Control rats administered orally with β-caryophyllene (200 mg/kg b.wt/day) once in a day, orally for 30 days.

Blood was collected after 30 days, and the animals were subjected to overnight fasting and perfused with physiological saline while anaesthetized with sodium thiopentone (40 mg/kg b.wt), and skeletal muscle was torn out to assess various parameters.

## Fasting blood glucose (FBG):

After overnight fasting, blood glucose was measured using On-Call Plus blood glucose test strips (ACON Laboratories Inc., USA). Blood was obtained by pricking the rats’ tail tip, and the results were reported in mg/dl.

## Lipid peroxidation and reactive oxygen species:

The technique of Devasagayam and Tarach [14] and was used to measure lipid peroxidation (LPO).The sample's malondialdehyde (MDA) concentration is measured in nmoles of MDA produced per minute per milligramme of protein. The spectrophotometric technique of Pick and Keisari [15] was used to measure hydrogen peroxide production, which was expressed as µmoles/min/mg protein. The generation of hydroxyl radicals (OH*) was measured using the Puntarulo and Cederbaum [16] technique and represented as µmoles/min/mg protein.

## Protein carbonyls Assay

The carbonyls in proteins were quantified using the dinitrophenylhydrazine (DNPH) reagent and a spectrophotometric technique published by Reznick and Packer [17]. At 370 nm, the absorbance was measured. The findings were calculated using a molar extinction coefficient of 22,000 M-1 cm-1 and represented as nanomoles of carbonyl groups per milligram of protein.

## Antioxidant enzymes

The superoxide dismutase (SOD) activity was measured using the Marklund and Marklund [18] technique and the findings were reported in units per milligram of protein. Catalase activity (CAT) was determined using Sinha's [19] technique, with the findings reported in units/mg protein. The activity of glutathione peroxidase (GPx) was measured using the Rotruck et al. [20] technique, and the results were reported as mg glutathione utilized/min/mg of protein.

## Skeletal muscle glucose metabolic parameters:

The technique of Nevado et al [21] was used to assess glucose absorption using 14C-2-dexoyglucose. The results are expressed as 14C-deoxyglucose uptake counts per minute (CPM) per 100 mg tissue. The technique of Muthuswamy *et al* [22] was used to assess glucose oxidation using 14C-glucose. CPM of 14CO2 released/100 mg tissue is used to represent the results. Glycogen content was determined using the Hassid and Abraham technique [23]. The quantity of glycogen concentration is expressed in mg/gram of wet tissue.

## Statistical analysis:

Using computer-based software, the data were analysed using one-way analysis of variance (ANOVA) and Duncan's multiple range test to determine the significance of individual differences between the control and treatment groups (Graph Pad Prism version 5).The significance of Duncan's test was determined at the level of P<0.05.

## Results:

## β-Caryophyllene modulates oxidative stress markers in type-2 diabetic adult male rats

**Figure 1:**
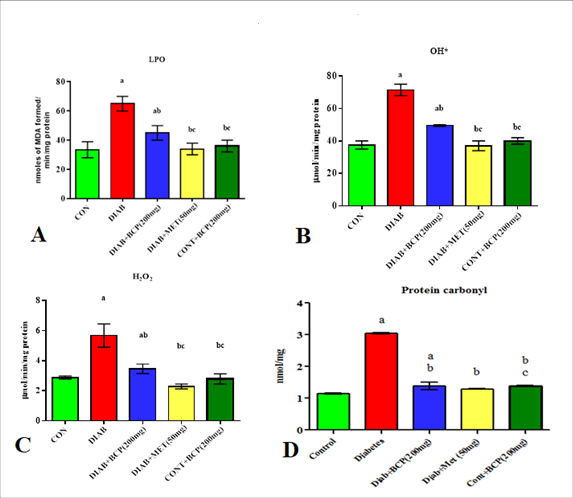
Effect of β- caryophyllene on oxidative stress marker in skeletal muscle of Experimental rats. Each bar represents Mean ± S.E.M of 6 animals. a- compared with control; b - compared with diabetic control rats; c- compared with 200mg/b.wt β-Caryophyllene. Significance was considered at the levels of p<0.05.

In skeletal muscle of diabetic rats, lipid peroxidation (LPO) (Figure 1A), hydroxyl radical (OH) (Figure 1B) and hydrogen peroxide (H2O2) (Figure 1C), and as well as protein carbonyl (Figure 1D) were considerably higher than in control rats. β- Caryophyllene significantly reduced lipid peroxidation, hydrogen peroxide, hydroxyl radical and Protein carbonyl levels.

## β-Caryophyllene enhances antioxidant enzymes in type-2 diabetic adult male rats:

**Figure 2:**
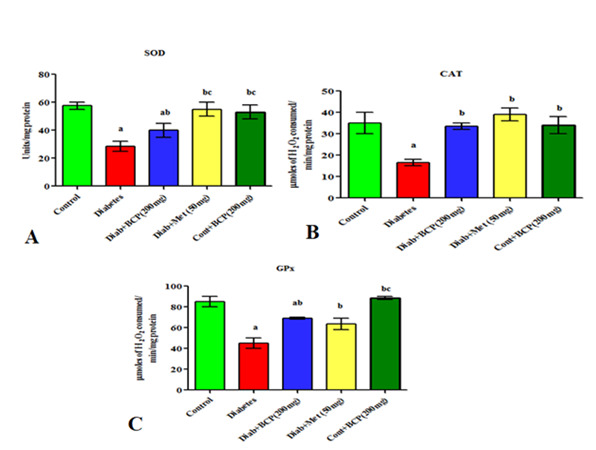
Effect of β- caryophyllene on antioxidant enzymes in skeletal muscle of Experimental rats. Each bar represents Mean ± S.E.M of 6 animals. a- compared with control; b - compared with diabetic control rats; c- compared with 200mg/b.wt β-Caryophyllene. Significance was considered at the levels of p<0.05.

In skeletal muscle of type-2 diabetic groups, superoxide dismutase (Figure 2A), catalase (Figure 2B) and glutathione peroxidase (Figure 2C) levels were significantly lower than in the control group. In comparison to the diabetic group, β- Caryophyllene effectively raised the amount of antioxidant enzymes.

## β-Caryophyllene improves 14C-2-deoxyglucose uptake and 14C-glucose oxidation in type-2 diabetic rats

**Figure 3:**
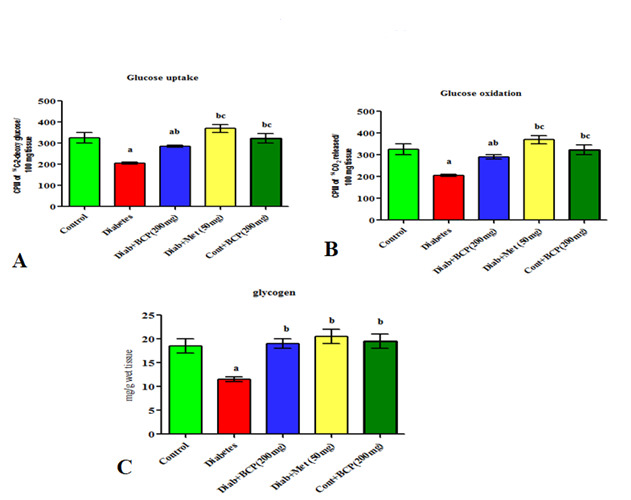
Effect of β-caryophyllene on 14C-2-deoxyglucose uptake, 14C-glucose oxidation and Glycogen content in skeletal muscle of Experimental rats. Each bar represents Mean ± S.E.M of 6 animals. a- compared with control; b - compared with diabetic control rats; c- compared with 200mg/b.wt β-Caryophyllene. Significance was considered at the levels of p<0.05.

In diabetic rat, glucose uptake and oxidation (Figure 3A and 3B) were considerably decreased. In diabetic rat, β- Caryophyllene enhanced glucose absorption and oxidation in the skeletal muscle as well as the conventional medication metformin. When comparing control and β- Caryophyllene treated rats, there was no significant difference.

## β- Caryophyllene improves Glycogen concentration in type-2 diabetic adult male rat

Effect of β- Caryophyllene on Glycogen concentration in type-2 diabetic was shown in figure: 3C when compared to control, type-2 diabetic rats showed a dramatically reduced glycogen concentration in skeletal muscle (Figure 3C). β- Caryophyllene therapy restored glycogen concentrations to a level comparable to metformin.

##  Discussion:

Diabetes is a long-term (chronic) illness that affects how your body converts food into energy and is becoming an important cause of morbidity and mortality. Body's resistance to insulin is one of the important factors contribute to the hyperglycemia in type-2 diabetes [24].

The idea that rats given a high-fat diet (HFD) develop insulin resistance and mimic the pathophysiology and clinical features of type 2 diabetes mellitus as it happens in people has been investigated in numerous experimental studies [25] Therefore, in our study, we fed rats a high-fat diet in an effort to cause type 2 diabetes. Given that interest in researching natural remedies is at an all-time high as a result of the irregularities in conventional treatments for diabetes, this model offers an ideal platform for evaluating antidiabetic agents. By evaluating different biological parameters, including oxidative stress, antioxidant capacity, and glucose metabolism in skeletal muscle, which is more visible during diabetes, our study demonstrated the antidiabetic activity of β -Caryophyllene.

High-fat diet and sucrose provoked hyperlipidemia leads to oxidative stress through increased ROS production which causes lipid peroxidation and membrane damage that promotes diabetic complications [26]. An imbalance between the production of reactive oxygen species (ROS) and the antioxidants in the cell is called oxidative stress. Hyperglycemic conditions result in the increased production leads to oxidative stress which promotes systemic insulin resistance [27]. β- Caryophyllene exhibits enhanced antioxidant activity in many experimental studies in both in vitro and in vivo. The present study evidenced that β- Caryophyllene treatment considerably decreased the MDA levels; a end product of lipid peroxidation, decreased the protein carbonyls; an end product of protein oxidative stress, as well as it also significantly reduced the levels of hydrogen peroxide and hydroxyl radicals. And diabetic rats treated with β-Caryophyllene showed an increased level of enzymatic antioxidants such as SOD, CAT and GPx in skeletal muscle of high fat diet and fructose induced type-2 diabetic rats. These findings elucidated that β-Caryophyllene has beneficial and protective role against HFD induced oxidative stress in diabetic rats, as a minimum in part, through attenuating lipid peroxidation and improving free radical scavenging activity [28].

Insulin resistance in skeletal muscle is the primary defect before the β-cell dysfunction and hyperglycemia [29]. Insulin activates the receptor tyrosine kinase when it binds to its receptor (IR), which in turn phosphorylates and recruits other IRS proteins. Tyrosine-phosphorylated IRS operates as PI3K binding sites, and when Akt/protein kinase B is activated, more intracellular GLUT4 is transported to the plasma membrane. The activation of the IRS/PI3K/Akt pathway facilitates glucose uptake by the skeletal muscle cells [30]. During insulin resistance conditions such obesity, hypertension, and type 2 diabetes, insulin-mediated glucose transport is reduced in the skeletal muscle.

This is due to impairment in the expression and functionality of the insulin signaling pathway [31]. In the present study, high fat diet-fed rats showed impairment in glucose uptake and oxidation. This is due to decreased level of GLUT4 in the plasma membrane as a result of impaired insulin signaling pathway which may be responsible for the elevated blood glucose in diabetic rats [32]. Treatment with β- Caryophyllene significantly increased the glucose uptake and oxidation which may be consequence of restored insulin signaling molecule thereby increased GLUT4 translocation of cytosol to plasma membrane. The concentration of glycogen was also significantly decreased in the skeletal muscle of diabetes induced rats when compared with control rats which is may be due to impairment in the process of glycogenesis as a result of diminished Akt phosphorylation at Thr308 which is essential event in the activation of glycogen synthase [33] Upon treatment with β- Caryophyllene the glycogen content was restored to the normal level.

## Conclusion:

The results of the present study elucidated that administration of β- Caryophyllene significantly improved glycemic status through increasing glucose uptake and oxidation by improving insulin signaling in skeletal muscle of diabetic rats. It also alleviates oxidative stress, improves antioxidants in diabetic rats. Hence, β- Caryophyllene can be used as one of the potential drug for the management of type-2 diabetes. Further studies on the effect of β- Caryophyllene on insulin downstream signaling molecules need to be carried out to ascertain its potential.
